# HLA-C Incompatibilities in Allogeneic Unrelated Hematopoietic Stem Cell Transplantation

**DOI:** 10.3389/fimmu.2014.00216

**Published:** 2014-05-19

**Authors:** Jean-Marie Tiercy

**Affiliations:** ^1^Transplantation Immunology Unit, National Reference Laboratory for Histocompatibility, Department of Genetics and Laboratory Medicine, University Hospital of Geneva, University of Geneva, Geneva, Switzerland

**Keywords:** HLA-C polymorphism, stem cell transplantation, incompatibilities, permissive mismatches, clinical outcome, HLA-C expression levels

## Abstract

An increasingly larger fraction of patients with hematological diseases are treated by hematopoietic stem cells transplantation (HSCT) from HLA matched unrelated donors. Polymorphisms of HLA genes represent a major barrier to HSCT because HLA-A, -B, -C and DRB1 incompatibilities confer a higher risk of acute graft-versus-host disease (aGVHD) and mortality. Although >22 million volunteer HLA-typed donors are available worldwide, still a significant number of patients do not find a highly matched HSC donor. Because of the large haplotypic diversity in HLA-B–C associations, incompatibilities occur most frequently at HLA-C, so that unrelated donors with a single HLA-C mismatch often represent the only possible choice. The ratio of HLA-C-mismatched HSCT over the total number of transplants varies from 15 to 30%, as determined in 12 multicenter studies. Six multicenter studies involving >1800 patients have reported a 21–43% increase in mortality risk. By using *in vitro* cellular assays, a large heterogeneity in T-cell allorecognition has been observed. Yet the permissiveness of individual HLA-C mismatches remains poorly defined. It could be linked to the position and nature of the mismatched residues on HLA-C molecules, but also to variability in the expression levels of the mismatched alleles. The permissive C*03:03–03:04 mismatch is characterized by full compatibility at residues 9, 97, 99, 116, 152, 156, and 163 reported to be key positions influencing T-cell allorecognition. With a single difference among these seven key residues the C*07:01–07:02 mismatch might also be considered by analogy as permissive. High variability of HLA-C expression as determined by quantitative RT-PCR has been observed within individual allotypes and shows some correlation with A–B–C–DRB1 haplotypes. Thus in addition to the position of mismatched amino acid residues, expression level of patient’s mismatched HLA-C allotype might influence T-cell allorecognition, with patients low expression-C alleles representing possible permissive mismatches.

## Introduction

HLA compatibility is a crucial parameter that influences the clinical outcome of hematopoietic stem cells transplantation (HSCT). Whereas it is technically simple to assess genotypic HLA compatibility in a related HSCT setting, the situation becomes more complex for unrelated donor selection. Here the developments in HLA typing technology have significantly contributed to improve the efficiency of unrelated HSC donor searches, resulting in a better recipient–donor HLA matching and a better prognostic for clinical outcome. In most transplant centers matching at HLA-A, -B, -C, DRB1, and DQB1 loci, a so-called 10/10 match, is the gold standard for unrelated donor selections (1–3). Although single DQB1 mismatches appear to be better tolerated than mismatches at other loci, they have an additive effect to A, B, C, DRB1 loci (1–4).

Historically, the recognition of HLA-C mismatches as *bona fide* transplantation antigens occurred a few years after the demonstration of the relevance of HLA-A, -B, DRB1 mismatches (5–7), because of the later developments in DNA-based analysis of HLA-C allelic polymorphism. Among HLA class I incompatibilities, a higher frequency of HLA-C compared to HLA-A, -B disparities has been disclosed, thus conferring a higher statistical power to the analysis of its role, possibly contributing to overestimate the impact of C-mismatches relative to disparities at other HLA loci. In any case large evidence points now to the clinical relevance of including HLA-C matching in the donor search algorithm.

The key role of HLA-C molecules as ligands of killer immunoglobulin-like receptors (KIRs) adds further complexity to a fair evaluation of the impact of HLA-C incompatibilities in HSCT. A large body of data pointing to the crucial role of HLA–KIR interactions in controlling the antileukemic activity of donor NK cells have been extensively reviewed elsewhere (8–10) and will therefore not be addressed here.

## Occurrence and Clinical Relevance of HLA-C Incompatibilities

Prospective and/or retrospective HLA typing of HSCT donor/recipient pairs showed that HLA-C mismatches represent 40–50% of all HLA class I and II incompatibilities (DPB1 not being considered) (Table 1). As determined in 11 different cohorts, a donor with a single HLA-C disparity had been selected in 13–31% of all transplants (Table 1). About 80% of HLA-C disparities concerned antigen mismatches (11–13). Thus HLA-C is by far the most frequent HLA mismatch, whereas the lowest rate of mismatches is found at locus DRB1. This probably reflects a more stringent priority given to DRB1 matching, less advanced technology for HLA-A, -B high resolution typing in clinical laboratories during the years 1990–2000, and more recent application of HLA-C prospective typing in the donor search algorithms (14).

**Table 1:** **Rate of HLA-C mismatches (MM) in different unrelated HSCT cohorts**.

Reference	C-MM/total nb MM (%)^a^	C-MM/total nb pat (%)^b^	Year TX^c^
(15)	156/412 (37.8)	156/973 (16)	1993–1998
(16)	–	749/1874 (40, incl. multiple MM)	1988–1996
(17)	85/248 single MM (34.3)	253/948 (26.7, incl. multiple MM)	1985–2003
(18)	34/74 (45.9)	34/144 (23.6)	1994–2003
(19)	29/69 (42)	29/214 (13.6)	1990–2003
(20)	91/187 (48.6)	91/334 (27)	1993–2003
(21)	478/985 (48.5)	478/2825 (16.9)	1988–2003
(22)	–	419/1361 (31)	
(11)	250/498 (50.2)^d^	250/1741 (14.4)	1999–2006
(12)	634/1524 (41.6)	634/2646 (24)	1997–2010
(23)	524/1037 (50.5)	524/3003 (17.4)	1993–2009
(24)	1016/2031 (50)^d^	–	1988–2009
(13)	895/1854 (48.3)^d^	895/6633 (13.5)	1988–2009

*^a^Number (nb) of patient/donor combinations with a single HLA-C mismatch (MM)/total nb of mismatches for HLA-A, -B, -C, DRB1, DQB1 loci*.

*^b^Number (nb) of patient/donor combinations with a HLA-C mismatch (MM)/total nb of patients*.

*^c^Time-frame of the HSCT included in the study (TX: transplantation)*.

*^d^Only 7/8 matched donors with single class I MM were considered*.

Ten clinical studies including >100 patients have specifically addressed the impact of HLA-C allele or antigen disparities and they all report a deleterious role of C-mismatches. As summarized in Table 2, six studies report a 20–100% increased risk of acute GVHD (11, 13, 15, 16, 21, 24). Out of the 10 studies analyzing overall survival, 8 report a 21–94% statistically significant increased mortality risk (11–13, 21, 24–26). Interestingly, the study that did not observe any impact of C-mismatches (assigned by retrospective typing) on survival concerns HSCT performed in 1993–1998 by the Japanese Marrow Donor Program (JMDP) (15). When JMDP patients transplanted in a later period (2000–2009) were analyzed the deleterious impact of C-mismatches became significant (23).

**Table 2:** **Hazard ratios for two outcomes in nine HSCT cohorts with single HLA-C mismatches**.

Reference	Nb pat	TCD/RIC	Year TX	Nb	aGVHD^a^	OS
				C-MM	HR (95% CI)	HR (95% CI)
(15)	1288	No TCD	1993–1998	156/272^b^	1.85 (1.42–2.41) *p* < 0.001	No impact
(17)	948	No TCD	1985–2003	24^c^	–	3.18 (1.74–5.82)
				61	–	1.32 (0.95–1.84)
(16)	1874	–	1988–1996	749	1.19 (1–1.41) *p* = 0.05	1.21 (1.06–1.38) *p* = 0.005
(25)	114 (CML)	32% TCD	1988–1999	24	–	1.94 (1.0–3.9) *p* = 0.06
(26)	111	100% RIC	2000–2004	33	–	1.85 *p* = 0.04
(21)	2825	36% TCD	1988–2003	382	1.6 (1.33–1.93) *p* < 0.001	1.22 (1.06–1.39) *p* = 0.004
		No RIC	
(11)	1933	35% RIC	1992–2006	168/189^d^	1.98 (1.5–2.62) *p* < 0.001	1.41 (1.16–1.70) *p* = 0.005
		No TCD			Grade III–IV	
(12)	3035	No TCD	1997–2010	620	–	1.35 (1.17–1.56) *p* < 0.001
		36% RIC	
(23)	751	No TCD/RIC	1993–1999	124	2.02 (1.27–3.20) *p* = 0.0029	0.96 (0.73–1.26) *p* = 0.77
	2252		2000–2009	386	1.51 (1.12–2.02) *p* = 0.0067	1.35 (1.15–1.59) *p* = 0.0003
					Grade III–IV	
(13)	6633	22% RIC	1988–2009	700^e^	1.72 (1.11–2.69) *p* = 0.02	1.43 (1.06–1.92) *p* = 0.02

*^a^Grade II–IV except for Ref. (16, 23)*.

*^b^C-mismatches and aGVHD risk: 156 single MM, 272 multiple MM*.

*^c^Single C-MM, low risk disease: 24 patients, high/intermediate risk disease: 61 patients*.

*^d^Antigen MM only (64 allele MM have no impact on GVHD/OS), 168 C-MM (aGVHD), 189 C-MM (OS)*.

*^e^Antigen MM*.

*TCD, T-cell depletion; RIC, reduced intensity conditioning*.

The evaluation of the relative importance of HLA-C mismatches may be influenced by differences in the frequencies of individual mismatches in different populations. For example, the C*03:03–03:04 mismatch accounted for 68.7% of C allele disparities in the recent CIBMTR study (13), but only 44.4% of the allele disparities detected in the JMDP study (28).

Furthermore, the relevance HLA-C *allele* versus *antigen* mismatches (11, 13, 21) may be biased by the nature of individual mismatches. Very few data are available concerning the relative frequencies of individual C-mismatches (13, 28, 29). Because of the very different HLA haplotypes in the Japanese population a significant fraction of the C-mismatches will not be encountered in patients of European ancestry. For example, allele mismatches within the C*07 allotype occur more frequently in patients cohorts of European ancestry, when compared to the JMDP study (28). As determined in the IHWS HSCT cohort C*03:03–03:04 mismatches accounted for 21/257 (8.2%) and 12/82 (14.6%) single (antigen and allele) HLA-C disparities, in JMDP and, respectively, non-JMDP patients (28). Of 257 HLA-C mismatched pairs identified in the non-JMDP patients, 172 (70%) were not found in the JMDP patient/donor pairs. Therefore the nature of individual mismatches strikingly differs in European and Asian populations.

## Relative Importance of Individual HLA-C Mismatches

In a first attempt from the JMDP to disclose individual HLA-C mismatches with higher clinical relevance, seven HLA-C allele mismatched combinations were reported to confer a higher risk of acute GVHD (29). Four amino acid residues at positions 9, 99, 116, and 156 of HLA-C molecules were significantly associated with acute graft-versus-host disease (aGVHD). A subsequent JMDP study showed that four specific HLA-C mismatches, C*01:02–14:02, C*08:01–01:02, C*14:02–03:04, and C*15:02–14:02 were associated with a lower risk of relapse (30), the latter being among the ones reported previously to confer a higher aGVHD risk (29).

New insights on the role of specific residues of HLA molecules in peptide-binding and TCR recognition (31) allowed a much better understanding of the T-cell alloreactive response to mismatched HLA antigens. The concept that specific residues could be essential in allorecognition led to a few clinical studies aimed at demonstrating the predominant role of discrete parts of the incompatible HLA molecules in clinical outcome (24, 32, 33).

Using a random forest statistical analysis 13 amino acid (aa) substitutions in the HLA class I peptide-binding pocket have been reported to be associated with increased mortality at day 100 in low/intermediate risk patients transplanted with HSC from a single HLA class I mismatched donor, with positions 9, 99, 116, and 156 being the most clinically relevant (33). HLA-C mismatch at position 99 was reported to be associated with increased TRM and a C-mismatch at position 116 with increased risk of acute GVHD.

When comparing individual C-mismatches at seven key residues in the peptide-binding site (PBS) that have been reported to be non-permissive substitutions marked differences are observed (Table 3). Strikingly, two of the most frequent mismatches exhibit no (C*03:03–03:04) or only one (C*07:01–07:02) amino acid (aa) difference at positions 9, 97, 99, 116, 152, 156, and 163, whereas all other common mismatches include a substitution at 3–6 positions. Whether this difference may allow classifying HLA-C incompatibilities as permissive/non-permissive, remains to be confirmed. It is however relevant to note that the C*03:03–03:04 mismatch did result in a negative or weakly positive CTL-precursor frequency (CTLpf) analysis (34, 35), and was therefore considered as a potential permissive mismatch. This is supported by a recent large scale study showing that 7/8 matched HSCT patients with a single C*03:03–03:04 mismatch had the same outcome as compared to 8/8 matched patients (13). On the other hand, two of the most frequent C-disparities differed by 5–6 key residues in the peptide-binding pocket: the C*15:02–14:02 mismatch in B51-positive patients and the C*04:01–12:03 mismatch in B35-positive patients. It is tempting to suggest that such mismatches could be considered as non-permissive.

**Table 3:** **Substitutions in the peptide-binding site (PBS) associated with outcome (3, 24, 32) in 13 frequent HLA-C mismatches as reported in Ref. (28)**.

HLA-C MM	Nb pat^a^	Residues in the PBS							Nb
		9	97	99	116	152	156	163	MM^b^
03:03–03:04	22	–	–	–	–	–	–	–	0
07:01–07:02	14	–	–	Y/S	–	–	–	–	1
15:02–14:02	13	Y/S	R/W	Y/F	L/S	–	L/R	–	5
04:01–16:01	10	S/Y	R/W	F/Y	F/S	E/A	R/Q	–	6
01:02–02:02	9	F/Y	W/R	C/Y	Y/S	–	R/W	–	5
05:01–07:04	8	Y/D	–	–	–	E/A	R/D	–	4
07:02–03:04	8	D/Y	–	S/Y	S/Y	A/E	–	T/L	5
14:02–01:02	7	S/F	–	F/C	S/Y	–	–	–	3
12:03–07:01	9	Y/D	–	W/R	–	E/A	W/L	–	4
02:02–15:02	7	–	–	–	S/L	–	W/L	E/T	3
12:03–04:01	5	Y/S	W/R	Y/F	S/F	–	W/R	–	6
03:04–04:01	5	Y/S	–	Y/F	–	–	L/R	L/T	4
01:02–15:02	5	F/Y	W/R	C/Y	Y/L	–	R/L	–	5

*^a^Number of patient/donor pairs*.

*^b^Number of mismatched residues in the PBS (out of the seven key amino acids reported to be associated with outcome)*.

## Recognition of HLA-C Incompatibilities by Alloreactive T Cells

In a first small scale study combining DNA typing and *in vitro* cellular assays such as the CTLpf analysis, the results suggested that HLA-C incompatibilities that were negative in an *in vitro* CTLpf assay may be of lower clinical relevance (5). It is well documented that single HLA class I allele mismatches, including HLA-C disparities, are efficiently recognized by CTLs (7, 35–42).

Based on CTLpf analysis of donor/recipient pairs with a single HLA-C incompatibility, alloreactivity was reported to be influenced by the number and position of the mismatched residues in the PBS (39). Negative CTLpf assays occurred more frequently in pairs with >9 aa differences compared to pairs with 0–5 aa differences in the α-helices and β-sheet. A model to predict cytotoxic T-cell alloreactivity was based on an amino acid mismatch score depending on the position and physicochemical properties of the mismatched residues (40). After testing the model on 171 9/10 and 168 10/10 matched HSCT patients, these authors found out that the aa mismatch score did not predict clinical outcome (41).

Infection- and vaccine-induced memory T cells have a high cross-reactive potential against allo-MHC molecules (43). Substitutions at key residues in the PBS (Table 3) are expected to modify the endogenous peptide repertoire bound to allo-HLA molecules, thereby increasing the cross-reactive potential of virus-specific memory T cells. Possibly multiple substitutions of key residues in the PBS (e.g., C*03:04–07:02) may increase the number of cognate ligands recognized by alloreactive T-cells (structural mimicry), as compared to mismatches with a single substitution such as C*07:01–07:02. Accurate modeling of peptide–HLA interactions (44) allowing to point to high risk non-permissive substitutions should contribute to a better evaluation of the permissiveness of HLA class I mismatched residues.

## HLA-C Expression Level and Alloreactivity

In the last couple of years, several studies have pointed to variability in HLA-C expression levels, as analyzed by RT-PCR and by DT9 antibody binding in heterozygous individuals. Such variability was shown to correlate with the antiviral response in HIV+ patients (45, 46). The association with HIV disease outcome was originally reported to be linked to a dimorphism at −35 kb upstream the HLA-C gene (27, 45). Subsequently, HLA-C expression was reported to be determined by a microRNA binding site dimorphism (263 indel) in the 3′UT region of HLA-C (47), and most recently by a *MIR148A* polymorphism affecting the microRNA miR-148a own expression and consequently the level of HLA-C expression in individuals carrying an intact miR-148a binding site (48).

Although the determination of HLA-C by DT9 antibody staining has the advantage to reflect *bona fide* cell surface expression it does not allow to dissect haplotype-dependant expression levels because the large majority of individuals are heterozygous. Even homozygous donors might show different HLA-C expressions levels that would depend on the extended ABDR haplotypes. This may account for apparent differences in expression levels reported previously. For example, HLA-C*07:02-positive (heterozygous) individuals were first classified among those with high expression allotypes (45), whereas it is found among the low expression allotypes in a study on African-American patients (46). Apparent inconsistencies are also noted when HLA-C allotypes were classified with high or low expression as based on DT9 antibody binding or on polymorphism in the 3′UTR miR-148 binding site. For example C*01:02 and C*04:01 are classified as low expression alleles based on the 3′UTR miR-148 binding site, but as high expression alleles based on DT9 binding. C*05:01 is classified as a high expression allele based on 3′UTR 263 indel, but is reported as low/intermediate based on DT9 binding. We have recently reported some evidence that HLA-C alleles can not be simply classified as high/low expression alleles, because significant interindividual variability was disclosed, at least for the C*03:04, C*04:01, C*05:01, or C*06:02 allotypes (49). Relevant correlation was found between HLA-C mRNA expression levels and the extended HLA-A, -B, DRB1 haplotypes. Under the hypothesis that differences in the level of expression might directly influence the CTL response, a simple test for quantifying allele-specific anti-HLA-C alloreactive T-cell responses or, indirectly, HLA-C recipient allotype expression levels, would help in donor selection and risk assessment before HSCT. Because HLA-C expression levels may impact both positive and negative thymic selection in the donor it is difficult to predict how this would translate in the donor T cell-mediated alloresponse against mismatched HLA-C antigens.

In summary, clinical studies point to a negative impact of single HLA-C mismatches in unrelated HSCT, with a 21–43% increased mortality risk. In the vast majority of the transplant centers unrelated donor search algorithms now take HLA-C compatibility into account. However, the diversity of HLA–B–C associations in specific haplotypes results in a rather high rate of HLA-C mismatches. The strength of the immune responses to HLA-C disparities may vary in different patient/donor combinations. HLA-C allorecognition will be impacted by the position of the mismatched residues on the HLA molecule, by the level of expression of the targeted mismatched allotype, and by the T-cell repertoire of the donor, which is modulated by the infectious history of each individual. Possible permissive mismatches may be assigned when no or very few substitutions occur at the key positions 9, 97, 99, 116, 152, 156, and 163 in the peptide-binding pocket, or in cases of low expression mismatched alleles, such as C*07:01/02 alleles. By analogy to findings concerning HLA-B incompatibilities (50), it may be anticipated that HLA-C disparities that involve residue(s) in the α3-domain (such as the C*02:02–02:29 mismatch) may be considered as permissive mismatches. This remains however to be demonstrated by functional *in vitro* assays, until clinical studies become large enough to provide sufficient statistical power for an analysis of α3-domain disparities. A better definition of such mismatches is required to increase the pool of acceptable donors, particularly for patients with rare HLA-B–C haplotypes.

## Conflict of Interest Statement

The author declares that the research was conducted in the absence of any commercial or financial relationships that could be construed as a potential conflict of interest.
